# Move – Mix It Up – Make It Fun: Summarizing and Visualizing Physical Activity Guidance

**DOI:** 10.2196/84225

**Published:** 2025-11-28

**Authors:** Julian Brummer, Florian Herbolsheimer, Mona Illmann, Duc Truong Nguyen, Karen Steindorf

**Affiliations:** 1 Division of Physical Activity, Cancer Prevention and Survivorship, German Cancer Research Center (DKFZ), Im Neuenheimer Feld 581, Heidelberg, 69120, Germany, 49 6221 42 2351; 2 Medical Faculty, Heidelberg University, Heidelberg, Germany

**Keywords:** physical activity, infographic, public health, slogan, AI, artificial intelligence

## Abstract

We derived a slogan summarizing contemporary physical activity guidance, “Move – Mix it up – Make it fun,” and created an infographic designed to illustrate these recommendations and support their use in physical activity campaigns.

## Introduction

Lifestyle factors like physical activity (PA) have a profound impact on human health. Hence, national and international public health authorities publish guidelines on these factors. Concise, easy-to-understand, actionable, and accurate public health messages derived from these extensive guidelines are desirable to improve their communication to the general public [1]. The simple three-line verse “Eat food. Not too much. Mostly plants” [2] sets an example here, providing a simplified summary of dietary guidance. To date, no similar slogan exists to summarize PA guidance. We aimed to close this gap and derive such a slogan for PA, based on, but not limited to, the 2020 PA guidelines of the World Health Organization [3]. In a second step, we aimed to design an appealing infographic to visualize the recommendations and support their use in PA campaigns.

## Methods

### Ethical Considerations

Ethics approval was not applicable as no human participants were involved in this research.

### Slogan

Our starting point was our own take on the most central public health messages arising from the scientific literature on PA for health. A first draft was discussed among the authors, aiming to improve its content and language. To complement our own ideas, we prompted various large language models (ie, Perplexity AI, ChatGPT, Claude AI) to generate ten ideas each for summarizing contemporary PA guidelines [4-6]. The suggestions of the large language models contained a wide range of summaries. We prioritized understandable, readable, memorable, and actionable messages primarily targeting adults and taking a meta-level perspective focusing on the essence instead of details. We also aimed to address the behavioral aspect of maintaining PA over time. Most importantly, the slogan had to be consistent with the literature on PA and health [3-7]. Taking these objectives into consideration, we finalized the slogan.

Textbox 1 shows the suggestions made by three different large language models when asked to summarize contemporary PA guidance to optimize the PA slogan. For Perplexity AI and ChatGPT, the following prompt was used: “Based on the attached reports, can you give me ten ideas on how to summarize the core guidance regarding physical activity? Please consider the following criteria for deriving your ideas: (1) three brief statements each, similar to the dietary guidance “Eat food. Not too much. Mostly plants,” (2) easy to understand, (3) concise, and (4) most importantly: scientifically accurate-please be very rigorous.” Due to input restrictions, for Claude AI, an adapted prompt without any attachments was used: “Based on the US, World Health Organization, and Canadian physical activity guidelines from 2018‐2020, can you give me ten ideas on how to summarize the core guidance regarding physical activity? Please consider the following criteria for deriving your ideas: (1) three brief statements each, similar to the dietary guidance “Eat food. Not too much. Mostly plants,” (2) easy to understand, (3) concise, and (4) most importantly: “scientifically accurate-please be very rigorous.”

Textbox 1. **Overview of suggestions made by the large language models.**

**Perplexity AI**

1. Move more. Sit less. Every bit counts.

2. Be active. Be regular. Mix intensities.

3. Aim for 150. Add strength. Break it up.

4. Start now. Progress gradually. Make it routine.

5. Any activity helps. More is better. Avoid none.

6. Move daily. Challenge muscles. Support balance.

7. Walk often. Lift things. Play safely.

8. Be active together. Enjoy movement. Keep it varied.

9. Reduce sitting. Increase steps. Find your way.

10. Move with purpose. Rest enough. Stay consistent.

**ChatGPT with reasoning feature**

1. Move 150. Lift 2×. Sit less.

2. Any move counts. More is better. Keep going.

3. 150 min. 75 min. Your choice.

4. Light steps. Big gains. Sit less.

5. Lift twice. Balance twice. Thrive.

6. Break up sitting. Move more. Live better.

7. Sleep well. Move well. Age well.

8. Kids on the move. Habits for life.

9. One bout today. One less drug tomorrow.

10. Go outside. Get stronger. Feel brighter.

**Claude AI**

1. Move daily. Build strength. Limit sitting.

2. Every minute counts. Strengthen twice weekly. Break up sitting.

3. Be active daily. Build muscle. Stand more.

4. Move more. Lift regularly. Sit less.

5. Start somewhere. Progress gradually. Stay consistent.

6. Sweat often. Strengthen muscles. Reduce screens.

7. Play daily. Build strength. Move frequently.

8. Exercise weekly. Strengthen regularly. Stand hourly.

9. Move your body. Challenge your muscles. Limit lounging.

10. Activity first. Strength second. Sitting last.

### Infographic

Aiming to create a visually appealing infographic that prioritized intuitive understandability and inclusivity, we consulted DALL-E to garner initial inspiration and an expert on visual communication of science who provided feedback on an early draft. Extensive work on various drafts among the multidisciplinary author group and retrieving the opinions of uninvolved individuals resulted in the final infographic.

## Results

**Figure 1.**
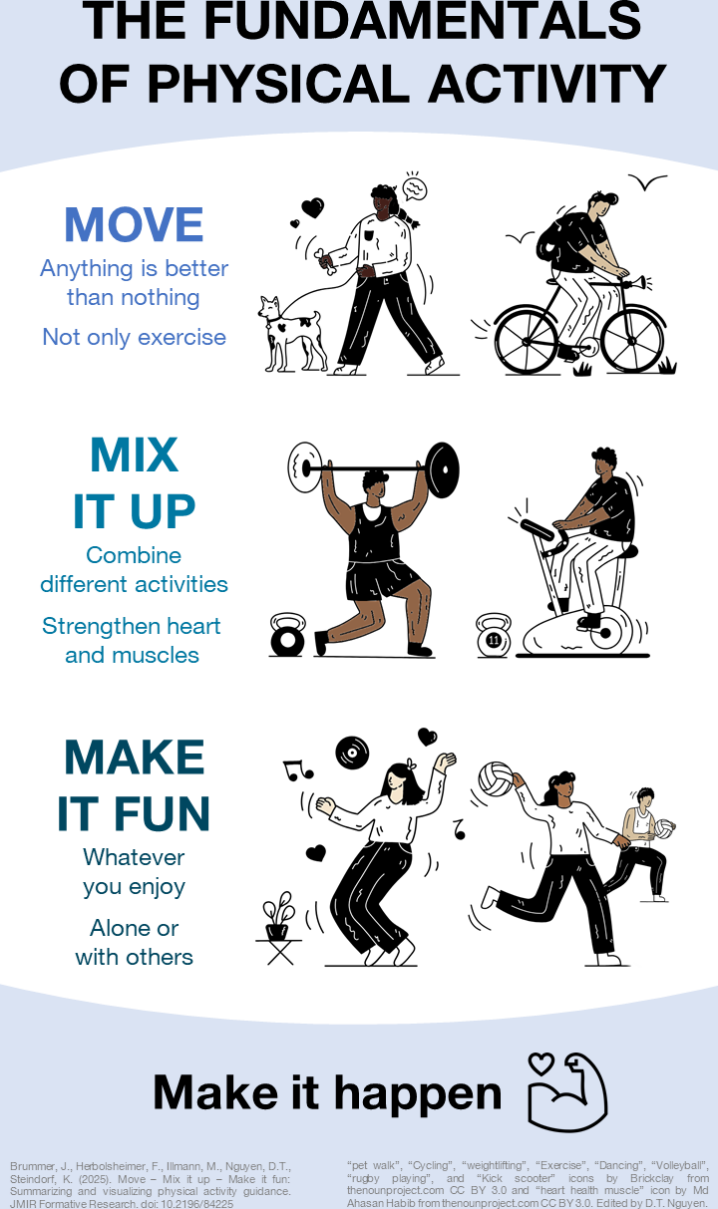
Infographic for the slogan “Move – Mix it up – Make it fun,” which was derived to summarize contemporary physical activity guidance by combining evidence from the scientific literature and the use of large language models to optimize its content and phrasing. The depicted infographic was designed to illustrate these recommendations and support their use in physical activity campaigns. The human drawings were sourced from The Noun Project originally [11] and were modified to depict more diversity and match the slogan; the attributions for the specific drawings are as follows: “pet walk”, “Cycling”, “weightlifting”, “Exercise”, “Dancing”, “Volleyball”, “rugby playing”, and “Kick scooter” icons by Brickclay from thenounproject.com CC BY 3.0 and “heart health muscle” icon by Md Ahasan Habib from thenounproject.com CC BY 3.0. Edited by D.T. Nguyen. The infographic is published under a Creative Commons Attribution 4.0 International License [12].

Ultimately, the discussions resulted in the final slogan: “Move**–**Mix it up**–**Make it fun.” Beyond meeting all of the above criteria—most importantly, being in line with current scientific evidence [3-9]—it allowed us to use an alliteration, which is a powerful linguistics tool [10]. The accompanying infographic is shown in Figure 1.

## Discussion

The present work presents a three-line verse that summarizes contemporary PA guidance—“Move **–** Mix it up **–** Make it fun.”—and an accompanying infographic that visualizes its contents.

Our slogan is in line with the current scientific literature on PA for health. Its first line, “Move,” encourages any type and amount of PA and therefore focuses on the great benefits that can be gained by going from no PA to some PA [3,4]. This acknowledges that health-promoting PA can also be done outside of the exercise domain (eg, active transport) [3], many different activities confer health benefits [3,8], and even light-intensity and lower volumes of PA can have substantial health benefits [3,6]. The second line gets more granular since it advises individuals to “Mix it up” and therefore include variation in their PA. This pertains primarily to the recommendation to engage in a combination of aerobic PA (eg, running, cycling) and muscle-strengthening activities (eg, resistance training) [3]. The third line, “Make it fun,” emphasizes the relevance of finding and engaging in activities and contexts (eg, those involving social interaction) that one personally enjoys. Unlike the preceding statements, this one is not a PA recommendation in itself, but rather a behavioral strategy designed to support the long-term maintenance of PA [9].

Our work illustrates the potential of combining large language models with human expertise for creating public health PA messaging. Yet, there are also limitations. First, our slogan is only one potential way of summarizing PA guidance; by taking a meta-level approach, specifics from PA guidelines were not incorporated. Second, this report presents an initial step. The effectiveness of the slogan and the accompanying infographic in changing PA attitudes, intentions, or behavior has not been tested yet; the present work serves as the basis for such future investigations.

After future evaluation, the slogan “Move **–** Mix it up **–** Make it fun.” may be used as a concise, memorable, easy-to-understand, and scientifically accurate tool to communicate actionable PA advice to the general public, for example in social media campaigns or related public health messaging—especially when longer messaging is not feasible or desired.

## Abbreviations

AI: artificial intelligence
PA: physical activity
